# Supplementary figures and images for: Cloning and Characterization of a Thermostable Endolysin of Bacteriophage TP-84 as a Potential Disinfectant and Biofilm-Removing Biological Agent
Source: Int J Mol Sci. 2022 Jul 9;23(14):7612. doi: 10.3390/ijms23147612 (PMC9325043; doi:10.3390/ijms23147612)

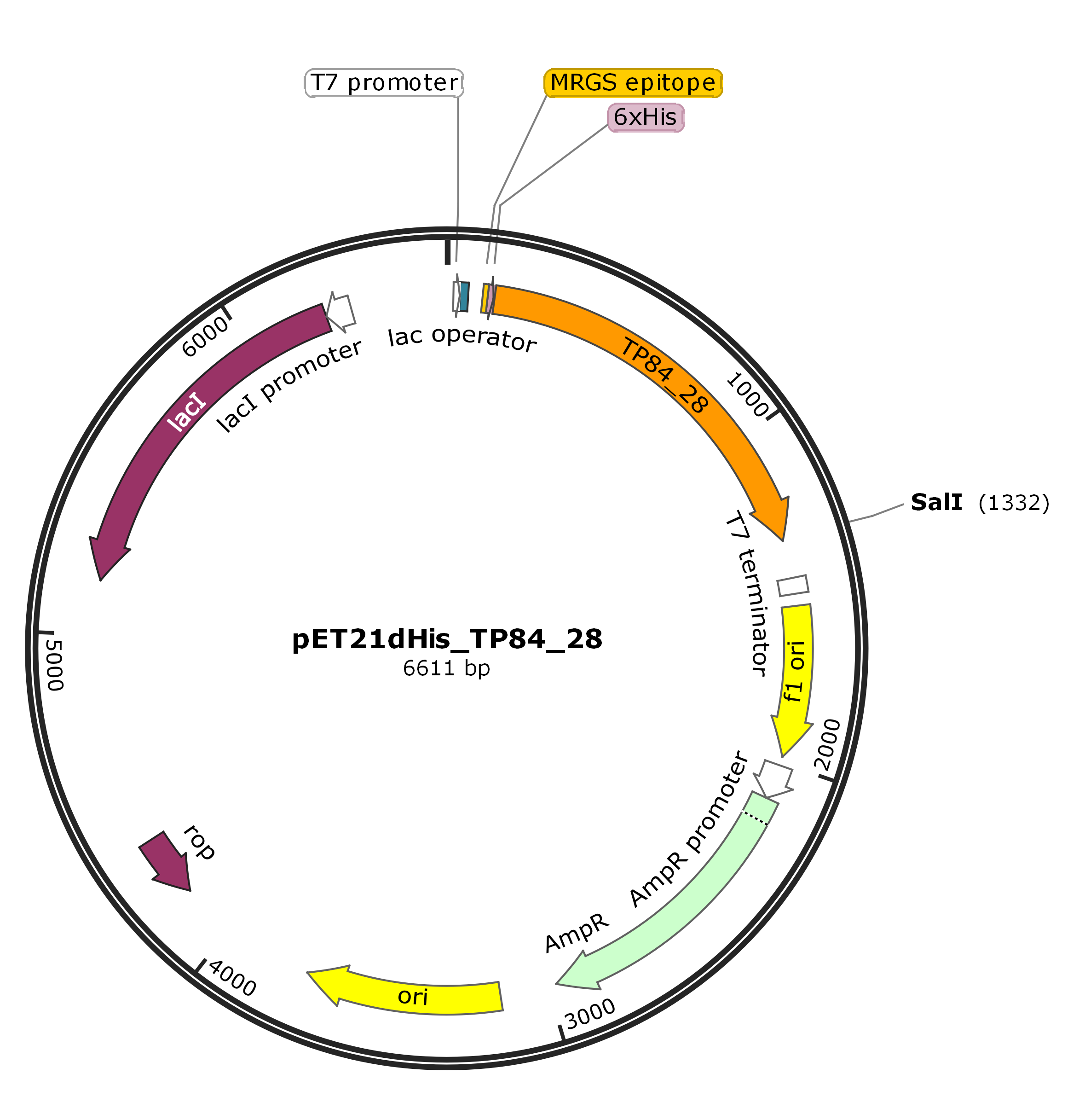

Supplement: Supplementary file 1 [file ijms-23-07612-s001.zip › S1_290422.tiff]

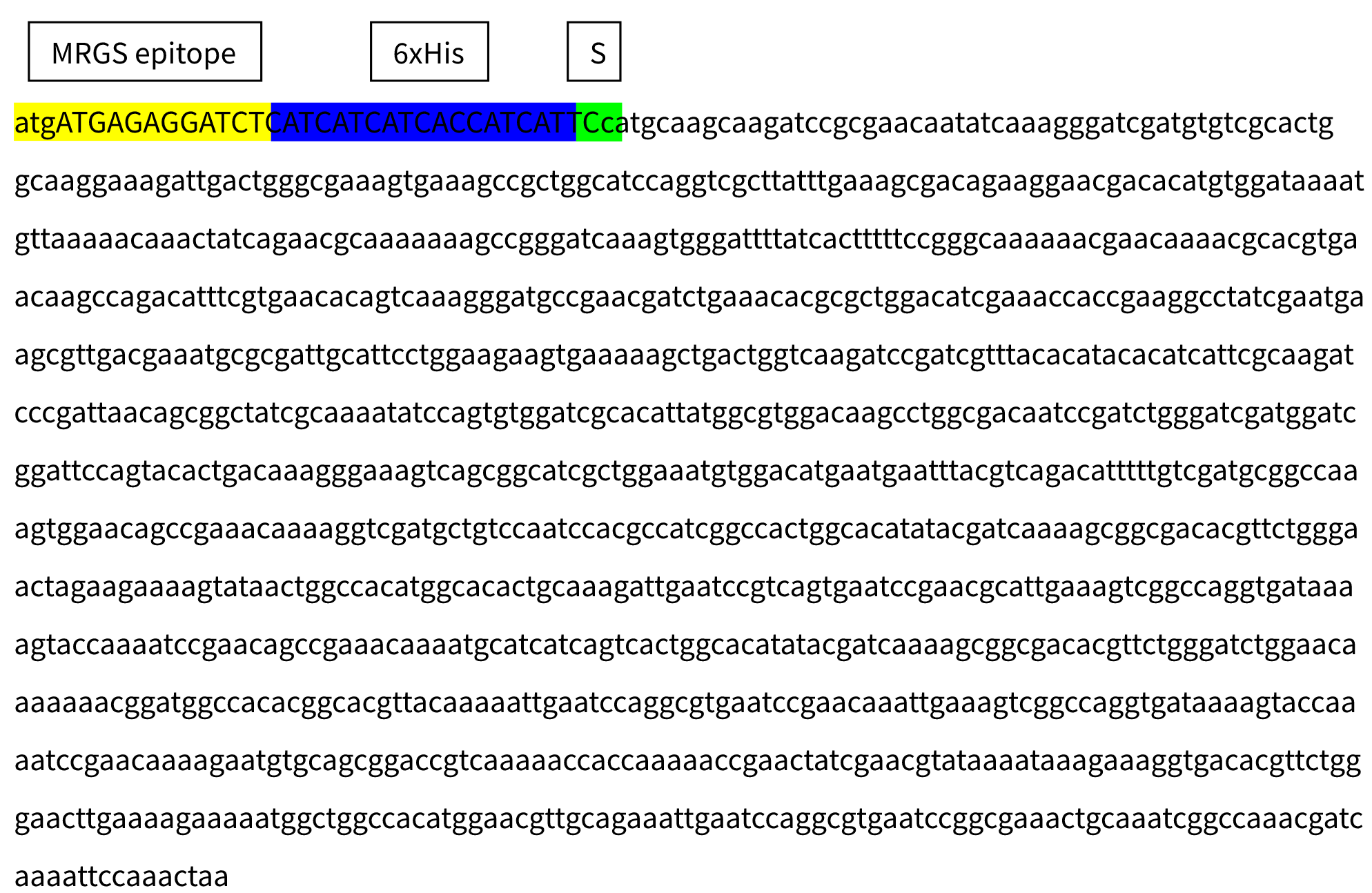

Supplement: Supplementary file 1 [file ijms-23-07612-s001.zip › S2_2204.tiff]

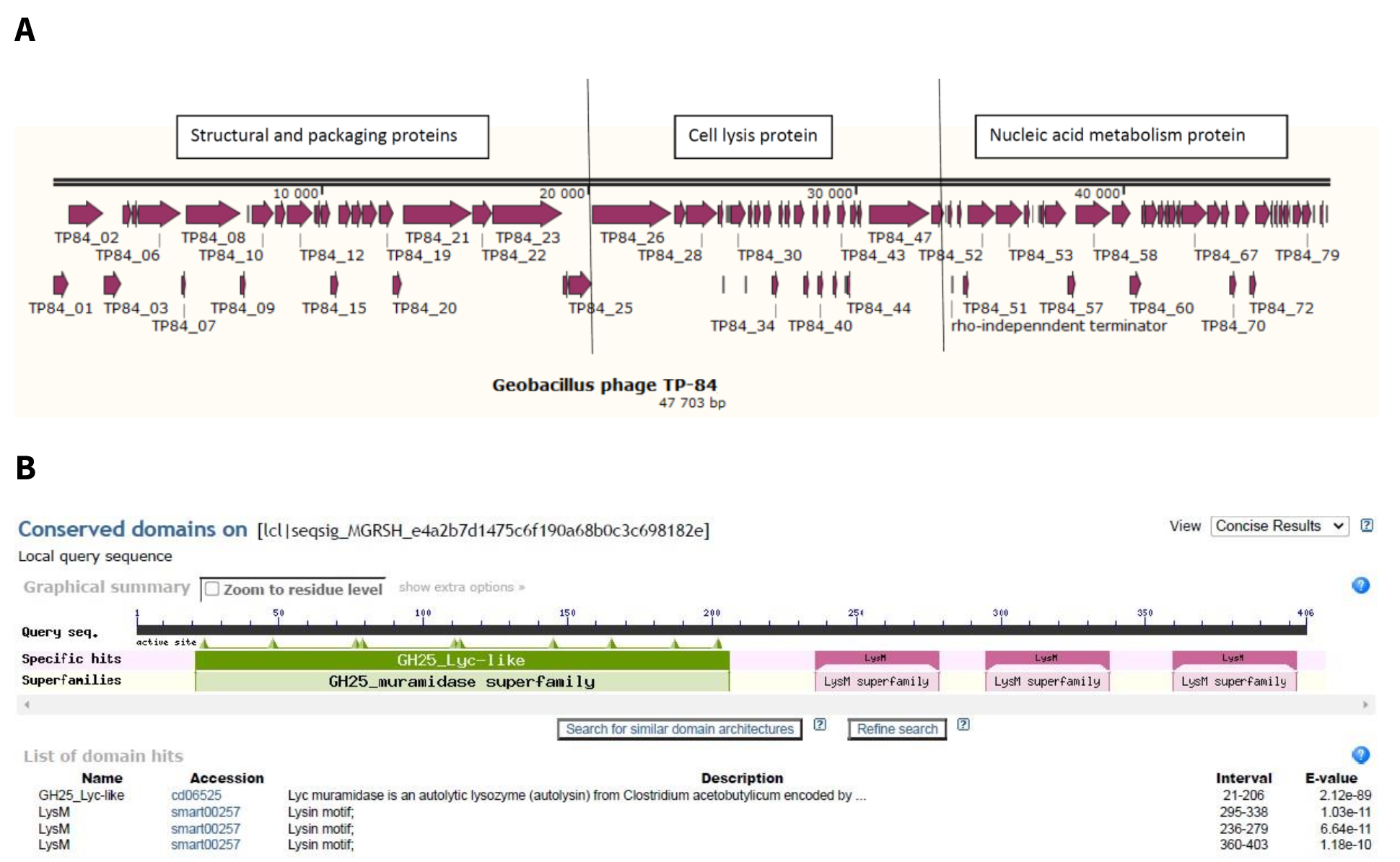

Supplement: Supplementary file 1 [file ijms-23-07612-s001.zip › S3_2204.tiff]

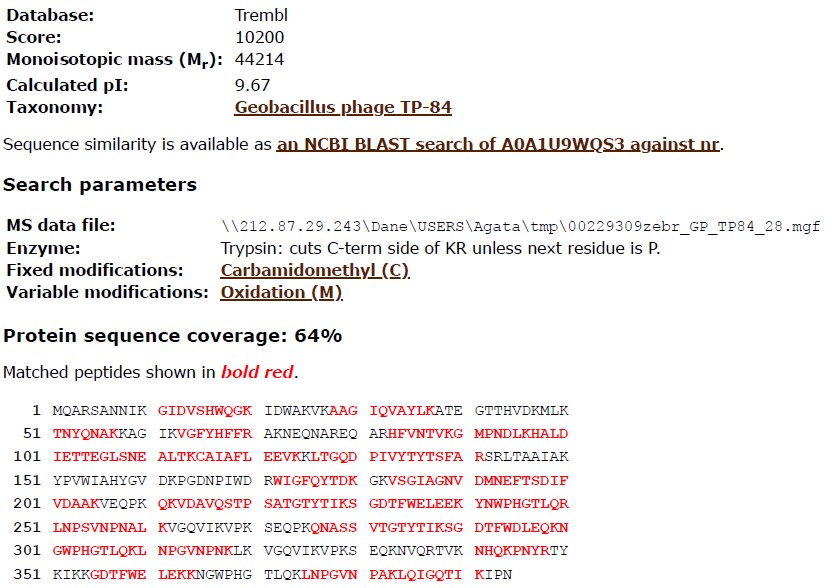

Supplement: Supplementary file 1 [file ijms-23-07612-s001.zip › S4_2904.jpg]
